# The Actual Status of Hospitals as COVID-19 Vaccination Clinics in China and Safety Monitoring of Inactivated Vaccine: A Cross-Sectional Study

**DOI:** 10.1017/dmp.2022.217

**Published:** 2022-08-26

**Authors:** Jin Huang, Mei-quan Zhang, Mei-zheng Huang, Gao-min Lin

**Affiliations:** 1 Department of Infectious Diseases, The Second People’s Hospital of Fujian Province, Fuzhou City, China; 2 Department of Respiratory Medicine, Fujian Geriatric Hospital, Fuzhou City, China

**Keywords:** COVID-19, inactivated vaccine, BBIBP-CorV, CoronaVac

## Abstract

**Background:**

The outbreak has had a devastating impact, and efforts are underway to speed up vaccination. The study’s objective was to describe the clinical characteristics of the coronavirus disease 2019 (COVID-19) vaccination clinic in the Second People’s Hospital of Fujian Province, China. Meanwhile, we monitored all the vaccine recipients to evaluate adverse reactions.

**Methods:**

A cross-sectional study was done at the COVID-19 Vaccination Clinic, the Second People’s Hospital of Fujian Province, China. We systematically collected Clinical data from the COVID-19 vaccination clinic between March 11 and November 11, 2021, including the type of vaccine, number of doses, gender, age, educational level, occupational category, adverse reactions, etc. Investigators will contact vaccine recipients by means of phone call or WeChat message to record the negative responses. Last, this report covers data through 8 mo, so it will be better to Evaluate the Safety of 2 inactivated COVID-19 vaccines from China (BBIBP-CorV [Beijing Institute of Biological Products, Beijing, China] and CoronaVac [Sinovac Life Sciences, Beijing, China]).

**Results:**

The results indicated that the Second People’s Hospital of Fujian Province received a total of 64,602 COVID-19 vaccines from March 11 to November 11, 2021, including 34,331 (53.14%) first doses, 29,245 (45.27%) second doses, and 1026 (1.59%) third doses. This study found the highest proportion in other personnel (38.69% at the first dose, 38.75% at the second dose, and 2.44% at the third dose), who were mainly retirees. People with higher levels of education are more likely to be vaccinated against COVID-19 during the early stages of vaccine rollout. In terms of age stratification, the highest proportion was found among people aged 18-49 (BBIBP-CorV: first dose 61%, second dose 62.6%, and third dose 76.8%; CoronaVac: first dose 66.1%, double dose 63.6%, and third dose 75.5%), followed by those over 60. The common adverse reactions were mainly local and systemic, and there were some differences between the 2 inactivated vaccines (*P* < 0.05).

**Conclusions:**

This is the first study to analyze the actual status of hospitals as COVID-19 vaccination clinics in China. The hospital has focused on vaccinating citizens and the initial rollout of vaccines to ensure any safety issues are identified. More citizens are willing to vaccinate in hospitals because of the uncertain safety of the available vaccines and adverse reactions. The good news is that vaccine-related severe adverse events have not been found in the hospital vaccination clinic. The Safety of BBIBP-CorV and CoronaVac is relatively high.

The coronavirus disease 2019 (COVID-19) outbreak was first reported in China in late 2019, which has caused the world to a standstill.^1^ According to WHO, as of November 11, 2021, there have been over 250 million confirmed cases globally, leading to at least 5 million deaths. Severe acute respiratory syndrome coronavirus 2 (SARS-CoV-2) is an enveloped single-stranded RNA virus with a 30 kb genome and 14 open reading frames, including 4 major viral structural proteins.^2^ Since the outbreak began, researchers worldwide have been racing to develop COVID-19 vaccines, with at least 198 vaccine candidates currently in preclinical and clinical development.^3^ Most COVID-19 vaccine candidates are based on the S antigen, such as viral vector, inactivated, subunit, nucleic acid-based DNA, and mRNA.^4^ To meet the emergency need for a vaccine, new vaccine research and development model has been proposed to shorten the COVID-19 vaccine development from 10-15 y to 1-2 y.^5^ Therefore, the vaccine’s safety needs to be closely observed and the related side effects carefully documented.

Due to the large population base in our country, the burden of vaccination in the Community Health Service Centers is heavy. China’s National Health Commission has rolled out a work plan to open additional hospitals to speed up the vaccination program. Therefore, the government has required hospitals to set up COVID-19 vaccination clinics. Another reason is that hospitals are chosen as vaccination sites for the safety of vaccines. The second People’s Hospital of Fujian Province is a comprehensive tertiary hospital, the first batch of hospitals to set up the COVID-19 vaccination clinic.

It is generally believed that the world will not return to its prepandemic normalcy until safe and effective vaccines become available and a global vaccination plan is successfully implemented.^6^ However, adverse events sometimes occur after large-scale vaccination of the COVID-19 vaccine. As the coronavirus is constantly mutating, the safety and effectiveness of the COVID-19 vaccine are also more concern.^7^ Sixty-four thousand six hundred two vaccine doses were injected by November 11, 2021, at the Second People’s Hospital of Fujian Province. This study presents the initial vaccination experience and analyzes the actual status of hospitals as vaccination clinics. In this study, the adverse events of BBIBP-CorV and CoronaVac were compared to understand the safety of inactivated vaccines.

## Methods

### Study Participants and Study Design

The COVID-19 vaccination data come from the Fujian Provincial Vaccination Management System. The study included the population who received their vaccine at the Second People’s Hospital of Fujian Province. From March 11 to November 11, 2021, the Second People’s Hospital of Fujian Province received 64,602 COVID-19 vaccines, including 34,331 first doses, 29,245-second doses, and 1026 third doses. All vaccine recipients voluntarily signed the informed consent forms for COVID-19 vaccination, and we obtained written parental consent for the minors before the study was begun. The study inquired about any adverse events to the COVID-19 vaccine and the timing of these side effects. We conducted two phone calls or WeChat: the first was a week, and the second was 3 wk after receiving the vaccine.

### Inactivated Vaccine

The primary type of vaccine is inactivated COVID-19 in Fujian Province, including BBIBP-CorV and CoronaVac. The inactivated COVID-19 vaccine is administered intramuscularly in 2 doses (0.5 ml each) before October, given 28-56 d apart. The third dose of booster injection is launched nationwide after October, and there is a 6-mo interval between the second and second doses. Lot numbers of BBIBP-CorV and CoronaVac vaccines are shown in Table 1.

**Table 1.** BBIBP-CorV and CoronaVac Lot No

CoronaVac (*n* = 39843)				BBIBP-CorV (*n* = 24759)			
Lot No.	dose	Lot No.	dose	Lot No.	dose	Lot No.	dose
202108044S	470	202104011E	999	2021020119	300	2021050959	1200
202104001E	1358	202104013J	1600	2021020144	492	2021050976	420
202104016F	1600	202104020E	601	2021040595	399	202105B0722	1000
202104027F	1600	202105031G	1140	2021040652	800	202105B0795	600
202105036G	1200	202105037B	1600	202105B0849	1400	202105B0883	960
202105038N	2400	20210 5040J	300	2021061441	900	202106B1202	2400
202105048J	1400	202105055K	1800	2021071630	810	2021071739	720
2021060410	2400	202106053N	1800	2021071902	300	202107B1667	800
202106062Z	1200	202106071L	1498	202107B1980	800	202107B1986	600
202106086F	2400	202106096F	1200	202108B2155	1200	202108B2303	1200
202106097H	2400	202106112G	1200	202108B2322	1200	202108B2694	400
202107085P	900	202107093M	600	202105C0174	1599	202106C0274	800
202107129E	900	202107119N	1500	202106C0254	1600	202106C0270	1200
202108137Y	1200	202108109P	900	2021040011J	200	202108J0464	459
202108148I	1500	E202103022	177	–	–	–	–

### Vaccination Clinic Configuration

Vaccination clinics should avoid being on the same floor or sharing access with potentially infected departments, such as the general door clinic, injection room, radiology department, infectious disease department (including fever clinic, bowel clinic, contagious disease wards), and laboratory. There should be the following functional areas: waiting room area, health inquiry area, registration area, informed notification area, vaccination area, observation area, abnormal reaction disposal area, and cold chain area. The process was arranged reasonably according to the sequence of waiting for vaccination, health inquiry, registration, notification, immunization, and observation. The entrance and exit of the vaccination area were set up separately, and the guidance signs were marked to achieve a 1-way flow and avoid cross-trips.

### Statistical Analysis

All analyses were done using GraphPad Prism (version 8.0). Continuous variables are expressed as median (IQR), and categorical variables are defined as count (percentage). The occurrence of adverse events was tested by chi-squared test, correction chi-squared test, or Fisher’s exact test between BBIBP-CorV and CoronaVac. A *P*-value of <0.05 was considered significant. All data were included in the analyses.

## Results

### Main Study Findings

**Figure 1.**
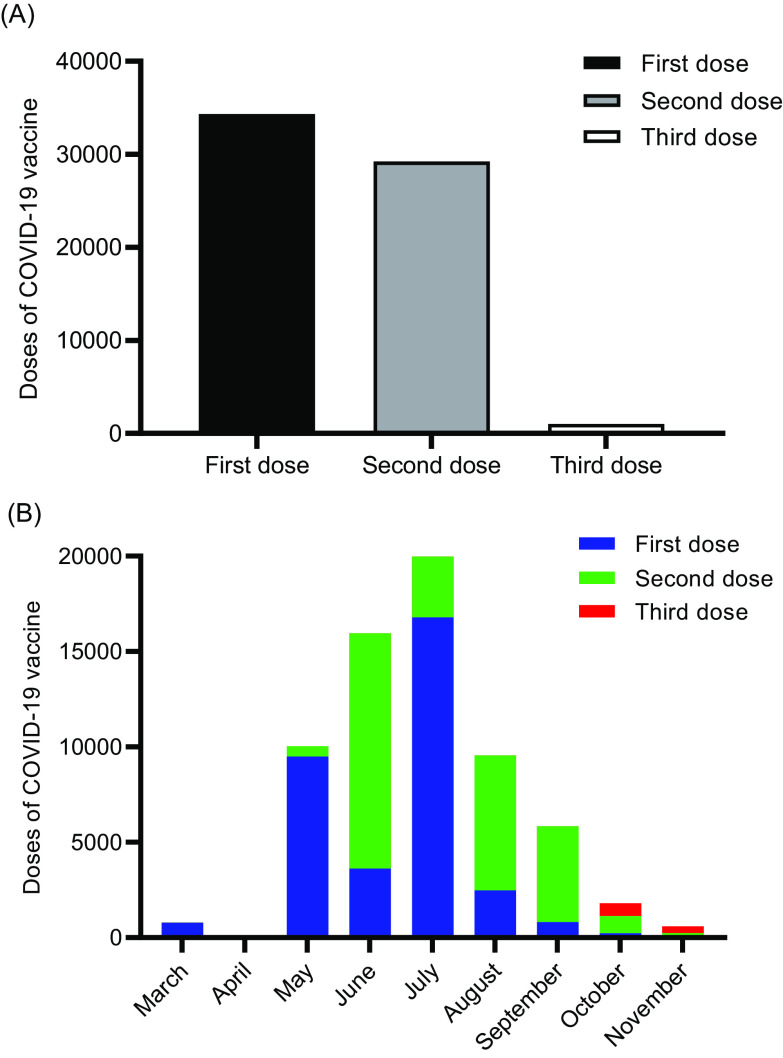
The doses of the COVID-19 vaccine from March 11 to November 11, 2021.

This study primarily focused on the COVID-19 vaccine BBIBP-CorV and CoronaVac. From March 11 to November 11, 2021, the Second People’s Hospital of Fujian Province received a total of 64,602 COVID-19 vaccines, including 34,331 (53.14%) first doses, 29,245 (45.27%) second doses, and 1026 (1.59%) third doses (Table 2). Figure 1 shows that vaccination is mainly concentrated from May to August, with the vaccination peak in July. According to the time distribution chart, the number of people decreased significantly from September to November.

**Table 2.** The doses of the COVID-19 vaccine from March to November 2021

Month	First dose *n* = 34331	Second dose *n* = 29245	Third dose *n* = 1026
March	789	2	0
April	49	1	0
May	9499	537	1
June	3622	12331	0
July	16790	3192	0
August	2476	7078	0
September	810	5028	0
October	232	896	678
November	64	180	347

### Data on the Occupational Classification

This study classified all COVID-19 vaccine recipients according to different occupations and found that other personnel accounted for the highest proportion (first dose 38.69%, second dose 38.75%, and third dose 2.44%), and such person was mainly retired employees. At the same time, we also found that the proportions of government officials and public institution personnel (9.91%), students in higher education schools (5.96%), and medical personnel (4.99%) are relatively high. Such people are more likely to receive the COVID-19 vaccine, which may be related to education. Due to the problematic COVID-19 situation worldwide, the number of people going abroad has decreased, so the vaccination rate of people going abroad (people going abroad on business 0.02%, people going abroad for personal reasons 0.07%, international students 0.17%, etc.) is at a low level (Table 3).

**Table 3.** Occupational classification data of COVID-19 vaccine recipients

Different professional	First dose (*n* = 34331)	Second dose (*n* = 29245)	Third dose (*n* = 1026)
Personnel working in places of detention	2(0.00%)	3(0.01%)	2(0.19%)
Customs and border control officers	4(0.01%)	6(0.02%)	0(0%)
People going abroad on business	7(0.02%)	6(0.02%)	8(0.78%)
International traffic crew	14(0.04%)	17(0.06%)	3(0.29%)
People going abroad for private reasons	23(0.07%)	26(0.09%)	3(0.29%)
COVID-19 isolation site staff	27(0.08%)	24(0.08%)	5(0.49%)
People in welfare institutions and nursing homes	28(0.08%)	20(0.07%)	2(0.19%)
International students	58(0.17%)	58(0.20%)	1(0.10%)
Handling, processing, and sales of imported products	71(0.21%)	37(0.13%)	0(0%)
Personnel dispatched to foreign labor service	122(0.36%)	103(0.35%)	1(0.10%)
Public security system, fire protection personnel	133(0.39%)	117(0.40%)	156(15.20%)
People engaged in agriculture, forestry, animal husbandry, and fishing	249(0.73%)	323(1.10%)	4(0.39%)
Community worker	263(0.77%)	194(0.66%)	40(3.90%)
Transportation, postal, and express industry personnel	428(1.25%)	336(1.15%)	42(4.09%)
Primary and secondary school students	505(1.47%)	728(2.49%)	0(0%)
Other workers in the health system	539(1.57%)	505(1.73%)	28(2.73%)
Educators	769(2.24%)	614(2.10%)	42(4.09%)
Basic social security personnel	1301(3.79%)	1205(4.12%)	104(10.14%)
Workers in labor-intensive industries	1371(3.99%)	1028(3.52%)	2(0.19%)
Medical personnel	1713(4.99%)	1651(5.65%0	275(26.80%)
Students in higher education schools	2045(5.96%)	1846(6.31%)	5(0.49%)
Government officials and public institution personnel	3403(9.91%)	2856(9.77%)	252(24.56%)
Domestic and unemployed persons	3582(10.43%)	2778(9.50%)	2(0.19%)
Service workers	4392(12.79%)	3432(11.74%)	24(2.34%)
Other personnel	13282(38.69%)	11332(38.75%)	25(2.44%)

*Note:* Other personnel were mainly retirees.

### Demographic Data of CoronaVac and BBIBP-CorV

**Figure 2.**
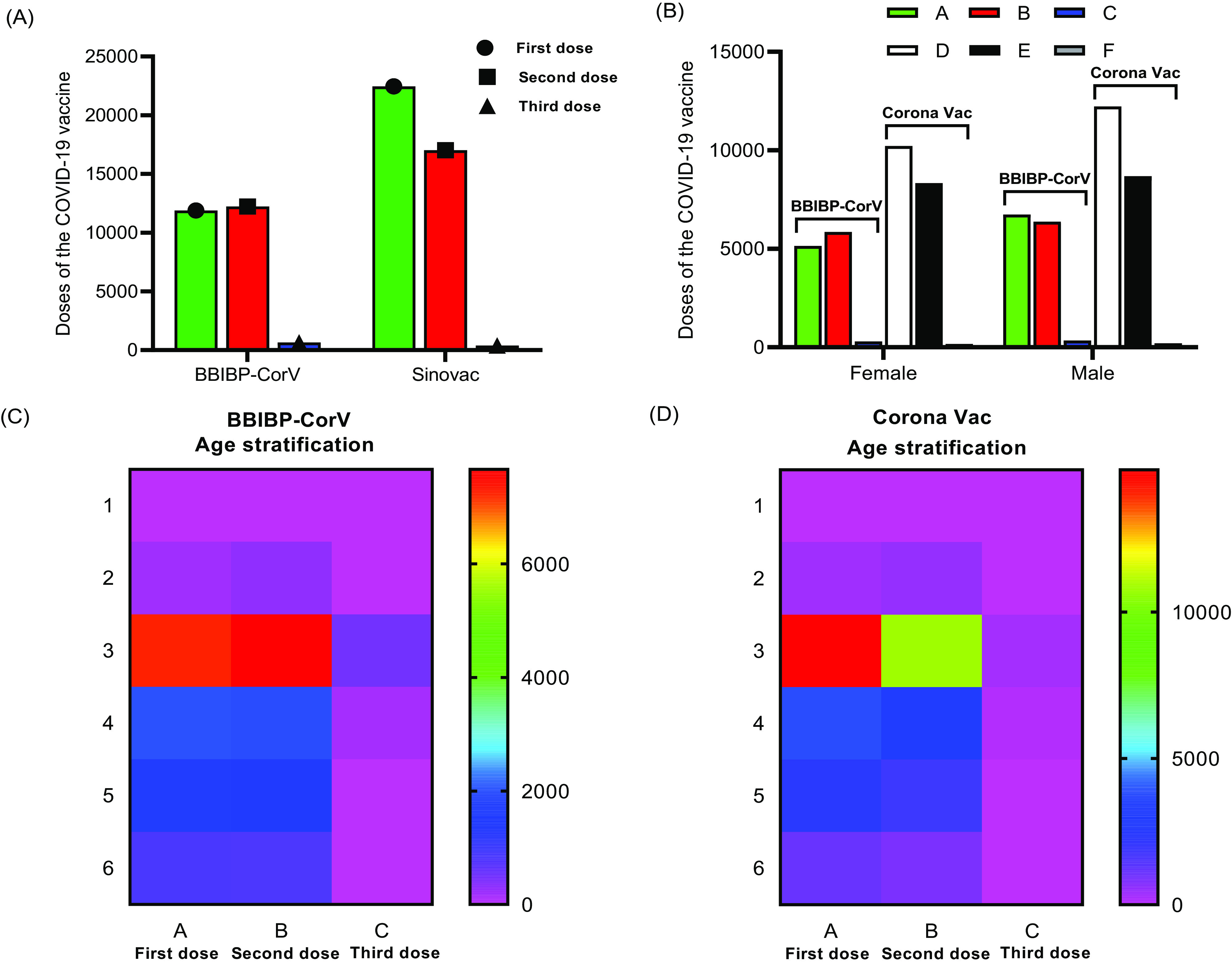
Demographic data of COVID-19 vaccine recipients. A, Population of BBIBP-CorV and CoronaVac in three doses. B, The distribution of the three injections of BBIBP-CorV (A: first dose; B: second dose; C: third dose) and CoronaVac (D: first dose; E: second dose; F: third dose) according to gender. C, Heat map of BBIBP-CorV according to age distribution. D, Heat map of CoronaVac according to age distribution.

Regarding the quantity of the 2 inactivated vaccines, the proportion of CoronaVac inoculation is higher (first dose 11883 vs 22448, second dose 12229 vs 17016), indicating that more people are willing to receive the Sinovac vaccine. In terms of age stratification, it is found that the proportion of people aged 18-49 is the highest (BBIBP-CorV: first dose 61%, second dose 62.6%, and third dose 76.8%; CoronaVac: first dose 66.1%, double dose 63.6%, and third dose 75.5%), and there are more people over 60 y old. The primary consideration is the vaccine’s safety, and it is more convenient to observe adverse events in the hospital. The low vaccination rate for people aged 3-11 and 12-17 is mainly related to the vaccination organized by the Health Commission. In terms of gender, there is no apparent difference between the 2 inactivated vaccines. In terms of education level, it is found that the proportion of doctor’s degrees, master’s degrees, and bachelor’s degrees is relatively high, which is related to the hospital’s central location (Table 4; Figure 2).

**Table 4.** Demographic data of two inactivated vaccines

	BBIBP-CorV [%(n/N)]			CoronaVac [%(n/N)]		
	First dose (*n* = 11883)	Second dose (*n* = 12229)	Third dose (*n* = 647)	First dose (*n* = 22448)	Second dose (*n* = 17016)	Third dose (*n* = 379)
**Age group (y)**						
3-11	0 (0%)	0 (0%)	0 (0%)	0 (0%)	0 (0%)	0 (0%)
12-17	172(1.4%)	299(2.4%)	0 (0%)	335(1.5%)	482(2.8%)	0 (0%)
18-49	7249(61%)	7660(62.6%)	497(76.8%)	14852(66.1%)	10806(63.6%)	286(75.5%)
50-59	2005(16.9%)	1952(16%)	143(22.1%)	3788(16.9%)	3016(17.7%)	93(24.5%)
60-69	1629(13.7%)	1501(12.3)	6(0.9%)	2375(10.6%)	1876(11.0%)	0 (0%)
≥70	828(7.0%)	817(6.7%)	1(0.2%)	1098(4.9%)	836(4.9%)	0 (0%)
**Gender**						
Female	5143(43.3%)	5858(47.9%)	301(46.5%)	10223(45.5%)	8327(48.9%)	169(44.6%)
Male	6740(56.7%)	6371(52.1%)	346(53.5%)	12225(54.5%)	8689(51.1%)	210(55.4%)
**Level of education**						
Doctor’s degree	642(5.4%)	513(4.2%)	33(5.1%)	1392(6.2%)	953(5.6%)	23(6.1%)
Master’s degree	1687(14.2%)	1357(11.1%)	88(13.6%)	4108(18.3%)	2280(13.4%)	60(15.7%)
Bachelor’s degree	3268(27.5%)	2727(22.3%)	160(24.7%)	6577(29.3%)	4152(24.4%)	103(27.3%)

### Safety of the BBIBP-CorV and CoronaVac

The primary outcome for safety was the occurrence of adverse events within a week after each vaccination. Those who had received the BBIBP-CorV and CoronaVac reported the adverse reactions based on their review of the body’s systems: local reactions, generalized reactions, respiratory reactions, musculoskeletal reactions, gastrointestinal reactions, neurological reactions, allergic reactions, cardiovascular reactions, and endocrine reactions.

Currently, serious vaccine-related adverse events have not been found in the hospital vaccination clinic. The common adverse reactions after vaccination are mainly local and generalized, which are relatively higher than other systemic reactions. Our follow-up found that BBIBP-CorV and CoronaVac had some differences in adverse reactions in different organ systems. BIBP-CORV was more likely to report musculoskeletal reactions than CoronaVac, such as muscle pain (2.36% vs 1.32%; *P* < 0.0001), joint pain (1.35% vs 1.11%; *P* < 0.0066), and muscle stiffness (0.49% vs 0.25%; *P* < 0.0001). BBIBP-CorV were more likely to report gastrointestinal reactions than CoronaVac, such as decreased appetite (3.37% vs 2.18%; *P* < 0.0001), nausea (1.37% vs 1.16%; *P* = 0.0200), and vomiting (1.08% vs 0.77%; *P* < 0.0001). BBIBP-CorV were more likely to report allergic reactions than CoronaVac, such as hives (1.65% vs 1.37%; *P* = 0.0039), nausea (0.96% vs 0.68%; *P* < 0.0001), and swelling in mouth (0.09% vs 0.03%; *P* < 0.0001). BBIBP-CorV were more likely to report blood pressure changes than CoronaVac (5.54% vs 4.23%; *P* < 0.0001). In addition, there was a significant difference in the rate of menstrual disorders between BBIBP-CorV and CoronaVac (0.13% vs 0.04%; *P* < 0.0001). But CoronaVac is more likely to report neurological reactions and respiratory reactions than BBIBP-CorV, such as cough (1.78% vs 1.57%; *P* = 0.0464), rhinorrhoea (0.56% vs 0.43%; *P* = 0.0224), dizziness (4.26% vs 3.03%; *P* < 0.0001), and numbness (1.23% vs 1.03%; *P* = 0.0207) (Table 5).

**Table 5.** Adverse event rate classified based on the review of various systems

Adverse event	BBIBP-CorV	CoronaVac	Chi-squared	*P*-value	Confidence interval
	Percentage reported [%(n/N)]	Percentage reported [%(n/N)]			
**Local reactions**					
Pain	2040(8.24%)	2235(5.61%)	170.9	<0.0001	1.42-2.41
Redness and swelling	911(3.68%)	932(2.34%)	98.90	<0.0001	1.45-1.77
Increase skin temp.	361(1.46%)	355(0.89%)	44.80	<0.0001	1.42-1.91
Itch	327(1.32%)	287(0.72%)	58.47	<0.0001	1.57-2.16
Induration	265(1.07%)	227(0.57%)	50.63	<0.0001	1.58-2.25
Rash	196(0.79%)	179(0.45%)	31.01	<0.0001	1.44-2.16
**Generalized reactions**					
Fatigue	2340(9.45%)	2940(7.38%)	87.36	<0.0001	0.61-1.38
Fever	807(3.26%)	1251(3.14%)	0.71	0.4001	0.94-1.13
Headache	708(2.86%)	697(1.75%)	88.46	<0.0001	1.48-1.83
Chills	305(1.23%)	211(0.53%)	95.05	<0.0001	1.96-2.79
Sweating	191(0.77%)	187(0.47%)	23.96	<0.0001	1.34-2.02
Lymphadenitis	104(0.42%)	28(0.12%)	91.62	<0.0001	3.99-9.09
**Musculoskeletal reactions**					
Muscle pain	584(2.36%)	526(1.32%)	97.53	<0.0001	1.60- 2.03
Joint pain	334(1.35%)	442(1.11%)	7.39	0.0066	1.05-1.41
Muscle stiffness	121(0.49%)	100(0.25%)	25.31	<0.0001	1.49-2.53
**Gastrointestinal reactions**					
Decreased appetite	834(3.37%)	870(2.18%)	83.48	<0.0001	1.42-1.72
Nausea	340(1.37%)	464(1.16%)	5.410	0.0200	1.03-1.36
Vomiting	267(1.08%)	307(0.77%)	16.44	<0.0001	1.92-1.66
Heartburn	250(1.01%)	283(0.71%)	16.73	<0.0001	1.20-1.69
Diarrhea	228(0.92%)	247(0.62%)	18.95	<0.0001	1.24-1.78
Abdominal pain	193(0.78%)	191(0.48%)	23.28	<0.0001	1.33-1.99
Constipation	178(0.72%)	88(0.22%)	92.38	<0.0001	2.53-4.24
**Respiratory reactions**					
Cough	389(1.57%)	709(1.78%)	3.96	0.0464	0.77-0.99
Sore throat	319(1.29%)	450(1.13%)	3.28	0.0701	0.99-1.32
Rhinorrhoea	106(0.43%)	223(0.56%)	5.21	0.0224	0.60-0.96
Nasal stuffiness	87(0.35%)	187(0.47%)	5.03	0.0249	0.57-0.96
Shortness of breath	79(0.32%)	16(0.04%)	80.90	<0.0001	4.66-13.95
Wheezing	52(0.21%)	12(0.03%)	49.94	<0.0001	3.82-13.45
**Neurological reactions**					
Dizziness	750(3.03%)	1697(4.26%)	63.39	<0.0001	0.64-0.76
Numbness	255(1.03%)	490(1.23%)	5.35	0.0207	0.71-0.97
Extremity weakness	151(0.61%)	287(0.72%)	2.76	0.0963	0.69-1.03
Reduced attention	111(0.45%)	84(0.21%)	28.62	<0.0001	1.61-2.82
Blurring of vision	32(0.13%)	20(0.05%)	11.86	0.0006	1.49-4.42
**Allergic reactions**					
Hives	409(1.65%)	546(1.37%)	8.31	0.0039	1.06-1.37
Eczema	238(0.96%)	271(0.68%)	15.44	<0.0001	1.18-1.68
Swelling in mouth	22(0.09%)	12(0.03%)	10.02	0.0016	1.51-5.73
**Cardiovascular reactions**					
Blood pressure changes	1371(5.54%)	1685(4.23%)	57.99	<0.0001	0.61-1.42
Palpitations	337(1.36%)	379(0.95%)	23.41	<0.0001	1.23- 1.66
Chest tightness	77(0.31%)	171(0.43%)	5.57	0.018	0.55-0.84
Chest pain	67(0.27%)	116(0.29%)	0.22	0.6331	0.68-1.25
Arrhythmia	57(0.23%)	64(0.16%)	3.95	0.0467	1.00- 2.03
Syncope	2(0.01%)	0(0.00%)	3.21	0.0728	0.74-1.34
**Endocrine reactions**					
Menstrual disorders	32(0.13%)	16(0.04%)	16.32	<0.0001	1.75-5.80

## Discussion

COVID-19 is still in the global pandemic stage, and vaccination is still the most economical and effective means to prevent and control the COVID-19 epidemic.^8^ Positive progress has been made in the global research and development of COVID-19 vaccines, and various vaccines have been approved for emergency use or conditionally launched in multiple countries and regions.^9^ However, its research and development cycle is short. Adverse reactions have been reported from time to time in the process of mass vaccination, and COVID-19 vaccination has not yet been widely recognized by society and people in various countries.^10^ It has been reported that the recipients of AstraZeneca’s adenovirus vector vaccine have experienced severe thrombosis. Many countries have also suspended vaccination of the vaccine, causing concerns from all walks of life.^11^

Based on the above results, vaccination is mainly concentrated from May to August, with the vaccination peak in July. Vaccine recipients were more likely to go to hospitals for COVID-19 vaccination in the early stages, mainly due to safety concerns. At the same time, it can be seen that the second dose has a downward trend. Early observations found that the elderly and those with many underlying diseases are more willing to go to the hospital to get the COVID-19 vaccine. Of note, 38.14% (24,639/64,602) of other personnel in the population are more willing to go to the hospital to get the COVID-19 vaccine, mainly elderly retirees.

Additionally, compared with other countries, decreased case reports in China might have contributed to increased hesitancy for COVID-19 vaccination during March and April. Physicians play a critical role in influencing vaccination decisions, and their recommendations are among the most vital factors of vaccine acceptability among citizens, and the COVID-19 vaccine will not be any different.^12^ Moreover, highly educated people are more likely to receive the COVID-19 vaccine, which may be related to education. This is in line with a study conducted in the United States.^13^ The SARS-CoV-2 has developed into a challenging situation worldwide, such as in India, the cases and deaths have markedly increased. Chinese people traveling abroad have decreased significantly, resulting in low vaccination rates.^14,15^

Regarding the number of inactivated vaccines, the proportion of CoronaVac vaccination is higher than BBIBP-CorV, and more people are willing to receive the Sinovac vaccine. In terms of age stratification, it is found that the proportion of people aged 18-49 is the highest, and there are more people over 60 y old. Therefore, it is recommended that all vaccinatees should be observed for at least 30 min after receiving the vaccine, following the Guidelines of the China CDC.

In the current study, no serious vaccine-related adverse events are found in the vaccination clinic of the Second People’s Hospital of Fujian Province. Most of the adverse events of the 2 types of inactivated vaccine recipients are mild to moderate, and most recipients can relieve themselves within 1-2 d after vaccination. Systemic reactions are diverse, including fatigue, fever, headache, chills, sweating, lymphadenitis, diarrhea, nausea, decreased appetite, blood pressure changes, unusual joint pain, etc. In contrast, local adverse effects include pain, redness, swelling, warmth, itching, induration, and rash. The inactivated vaccine is the most traditional and classic vaccine preparation, which has higher safety than other new vaccines.^16^ Comparative analysis shows that BBIBP-CorV and CoronaVac have some differences in the incidence of vaccine adverse reactions, and the overall safety of both is relatively high. For example, BIBP-CorV is more likely to report musculoskeletal reactions than CoronaVac, such as muscle pain (2.36% vs 1.32%), joint pain (1.35% vs 1.11%), and muscle stiffness (0.49% vs 0.25%). But CoronaVac is more likely to report neurological reactions and respiratory reactions than BBIBP-CorV, such as cough (1.78% vs 1.57%), rhinorrhea (0.56% vs 0.43%), dizziness (4.26% vs 3.03%), and numbness (1.23% vs 1.03%). Therefore, our observations show that BBIBP-CorV and CoronaVac have different adverse reactions in different organ systems. So, the health-care workers need to raise awareness of various adverse reactions to avoid serious consequences. Some studies have found other adverse effects, such as Bencharattanaphakhi and Rerknimitr reported 2 cases of CoronaVac-induced cutaneous vasculitis, a rare cutaneous adverse event after vaccination. However, cutaneous vascular inflammation has not been reported from the use of BBIBP-CorV.^17^ Of interest, no cases were reported from BBIBP-CorV, which is also based on the inactivated whole virus. Chuaychoosakoon et al. reported the first case of a SIRVA after a Sinovac COVID-19 vaccination, which occurred due to deep penetration and direction of the needle.^18^ The patient’s clinical symptoms improved after treatment with combined oral non-steroidal anti-inflammatory drugs and a short course of intravenous antibiotics.^19,20^ The inactivated COVID-19 vaccine BBIBP-CorV and CoronaVac are safe and satisfactorily tolerated at all tested dose levels in participants aged 3-17 y.^16,21,22^ There are differences in the incidence of adverse reactions between the 2 inactivated vaccines in certain systems, which provides guidance for patients with chronic diseases in choosing vaccines. However, it is unknown whether the vaccine’s viral particles or excipients are responsible as an antigen for such reactions.

Currently, a variety of COVID-19 vaccines are on the market around the world. Among them, BBIBP-CorV and CoronaVac produced in China are inactivated vaccines, different from nucleic acid and viral vector vaccines in terms of technical routes.^23^ For example, BNT162b2 (Pfizer-BioNTech) and mRNA-1273 (Modena) both use nucleic acid technology. The mRNA vaccine has more robust immunogenicity than traditional vaccines, but the inactivated vaccine may have milder reactivity.^24^ A prospective cohort study at the University of Hong Kong aimed to compare self-reported postvaccination adverse reactions between CoronaVac and BNT162b2. The results confirmed our hypothesis that CoronaVac had milder reactogenicity compared with BNT162b2. They also found that the risk of adverse responses 2 wk postvaccination is significantly lower among those receiving CoronaVac than BNT162b2.^25^

A comparative study in Brazil found CoronaVac’s response rate was 50.7%, AstraZeneca’s was 79%, and Pfizer’s was 91.5%. Results show that AstraZeneca has the best cost-benefit when prioritizing acquisition costs, while Pfizer is the most cost-beneficial when prioritizing the number of deaths.^26^ Against the historical strains, the efficacy of the BNT162b2 and mRNA-1273 vaccine was greater than 90% at 6 mo of follow-up after the second dose, while CVnCoV had lower effectiveness of 48%.^27^ BNT162b2 and CoronaVac vaccine in cancer patients were evaluated in a single-center, cross-sectional, and descriptive study at Bezmialem Vakif University Medical School in Turkey. This study showed no difference between BNT162b2 and CoronaVac in an intra-group comparison of patients vaccinated with the total dose.^28^

AZD1222 and Sputnik V are both adenovirus vector vaccines, which are 65%-91.6% effective against historical strains.^27^ The side effects of the AZD-1222 vaccine were more evident than the Sputnik V vaccine. The most common side effects of the AZD-1222 and Sputnik-V vaccine among Birjand health-care workers were injection site pain (62.1%), fatigue (43.9%), muscle pain (42.5%), and fever (40.6%). In this study, muscle symptoms, fatigue, and fever were common side effects of AZD-1222, consistent with our study.^29^ Iranian research comparing vector-based and inactivated vaccines found that the ChAdOx1 NCOV-19 group had higher severity of side effects than the other groups (BBIBP-CorV and GAME-COVID-VAC).^30^ A study in UAE showed that inactivated vaccine BBIBP-CorV (95%) and the mRNA vaccine BNT162b2 (98%) demonstrated protection against COVID-19 related hospitalizations from the Delta (B.1.617.2) variant.^31^ A comparison of different types of vaccines worldwide shows that inactivated vaccines have lower side effects and higher safety, but may be less effective than mRNA vaccines.

There are several limitations to our current study. The data for this study were mainly from 1 hospital and reflected limited vaccination status. The follow-up method is relatively simple and lacks objectivity, and the follow-up time is short (8 mo). The participants had limited racial and ethnic diversity compared with the general population. The sample size of this study was relatively small; thus, further studies with larger sample sizes are required to ascertain the safety profile. In addition, a multi-center evaluation of the vaccine from China is needed to examine the effectiveness of the COVID-19 vaccines.

## Conclusions

The high acceptance rate of the first dose is a positive phenomenon, and it can encourage future vaccine recipients to receive the second and third doses, irrespective of side effects. In the early stages, hospital vaccination clinics have provided more robust health-care facilities for citizens, so more people are willing to vaccinate in hospitals when the safety of vaccines is uncertain. The most common adverse reactions were mainly mild to moderate in severity, transient, or resolved in a few days. In conclusion, no severe side effects were observed in this study, and most of the adverse events of the 2 types of inactivated vaccine recipients are mild to moderate.

## Data Availability

The datasets used and analyzed in this study belong to our research team, and the data does not involve personal privacy information. The datasets are available from the corresponding author on reasonable request.

## Supplementary material

For supplementary material accompanying this paper visit https://doi.org/10.1017/dmp.2022.217. click here to view supplementary material
