# Supplementary material for: The Actual Status of Hospitals as COVID-19 Vaccination Clinics in China and Safety Monitoring of Inactivated Vaccine: A Cross-Sectional Study
Source: Disaster Med Public Health Prep. 2022 Aug 26:1–8. doi: 10.1017/dmp.2022.217 (PMC9588414; doi:10.1017/dmp.2022.217)

## Related files

**The COVID-19 vaccination original data come from the Fujian Provincial Vaccination Management System.**

**Fig.1** BBIBP-CorV and Corona Vac Lot No.


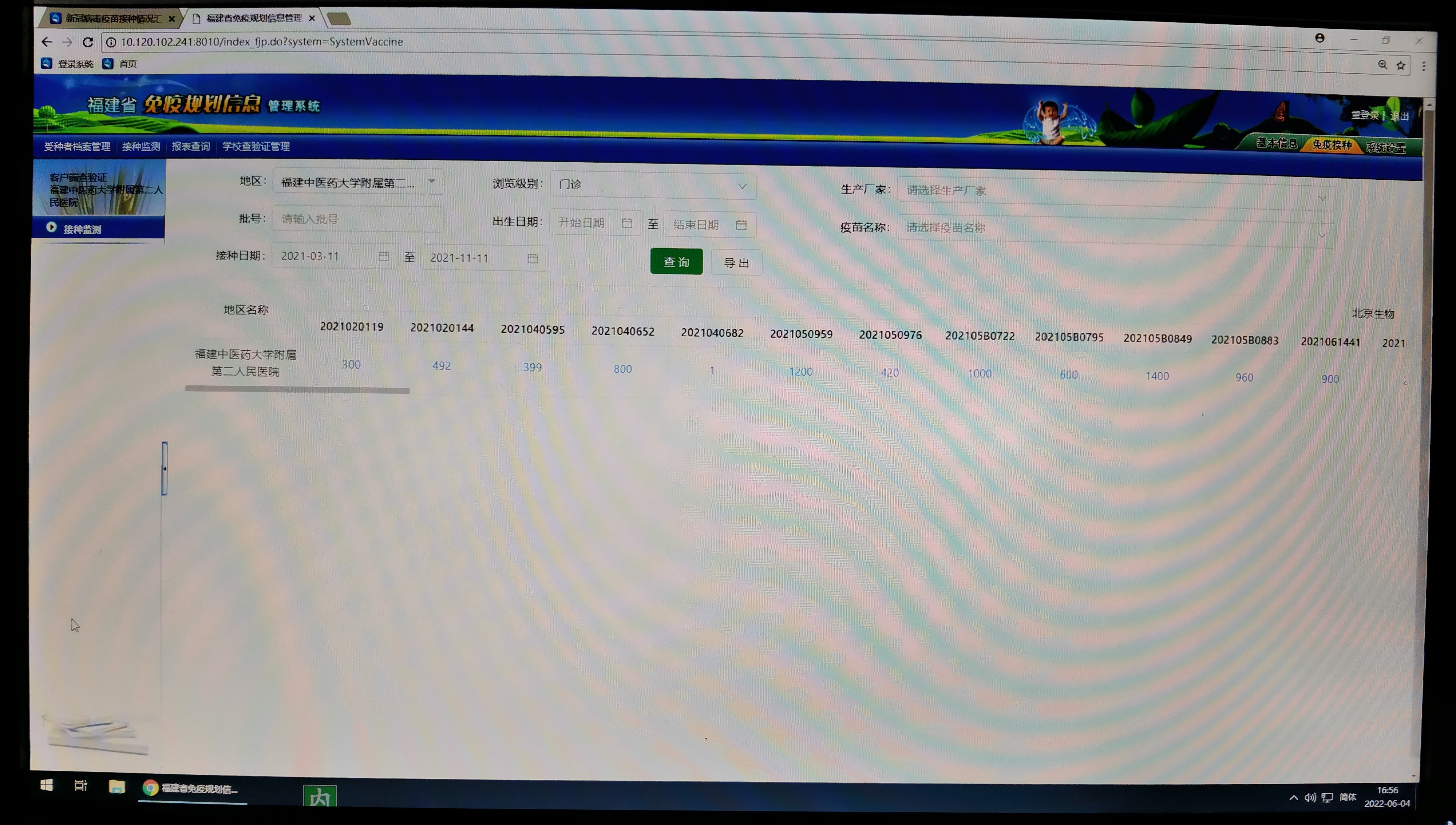

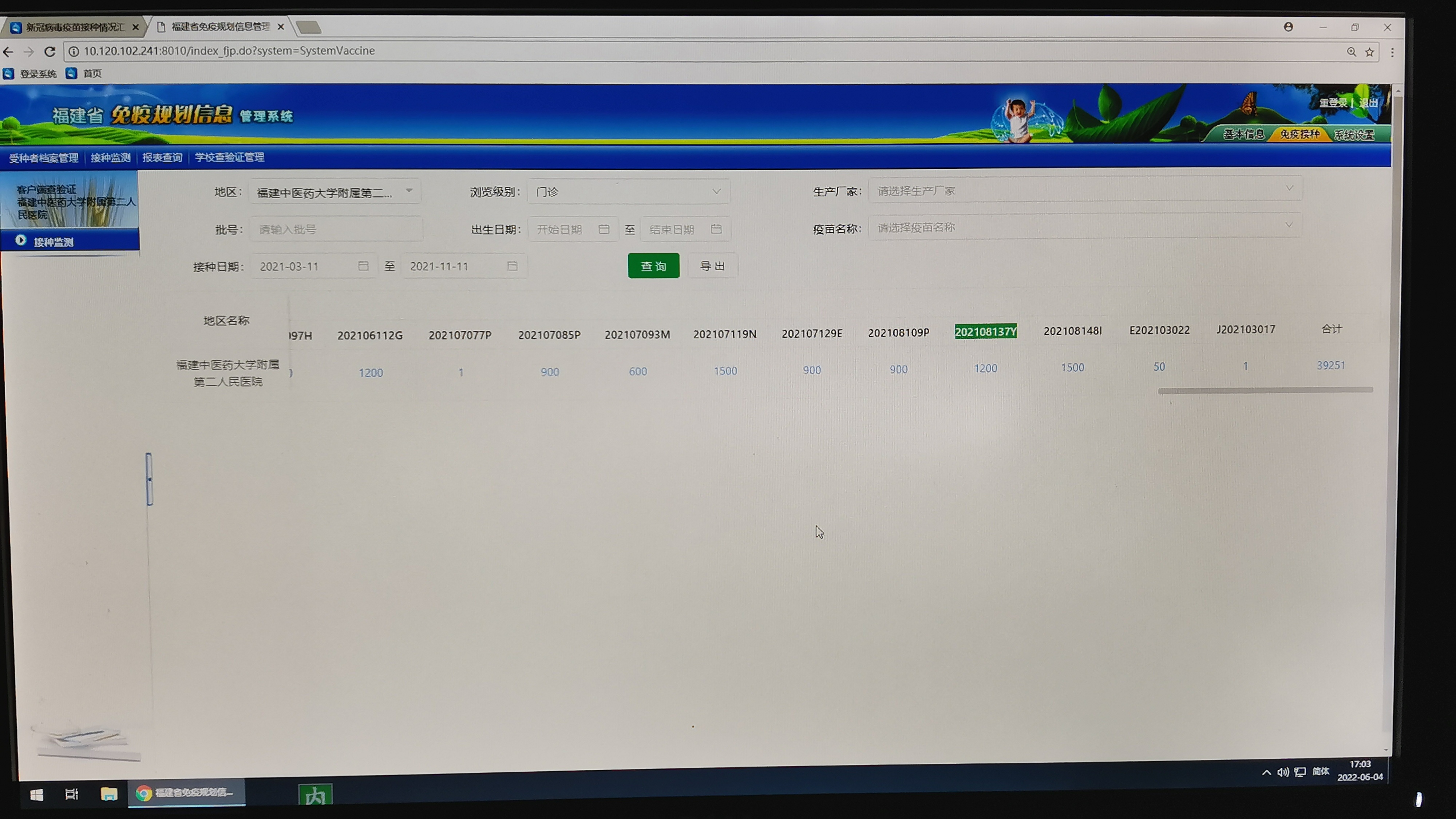

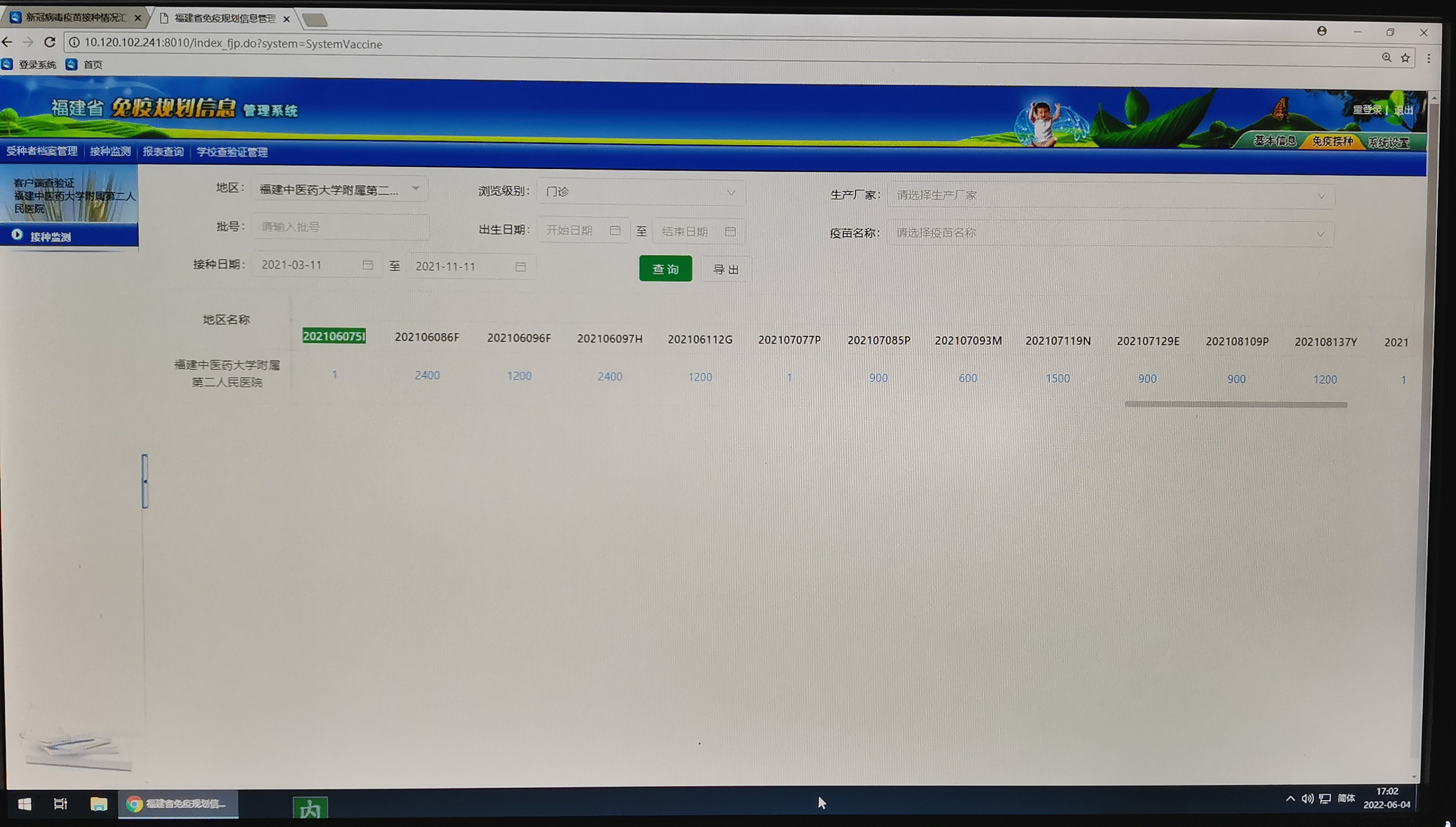

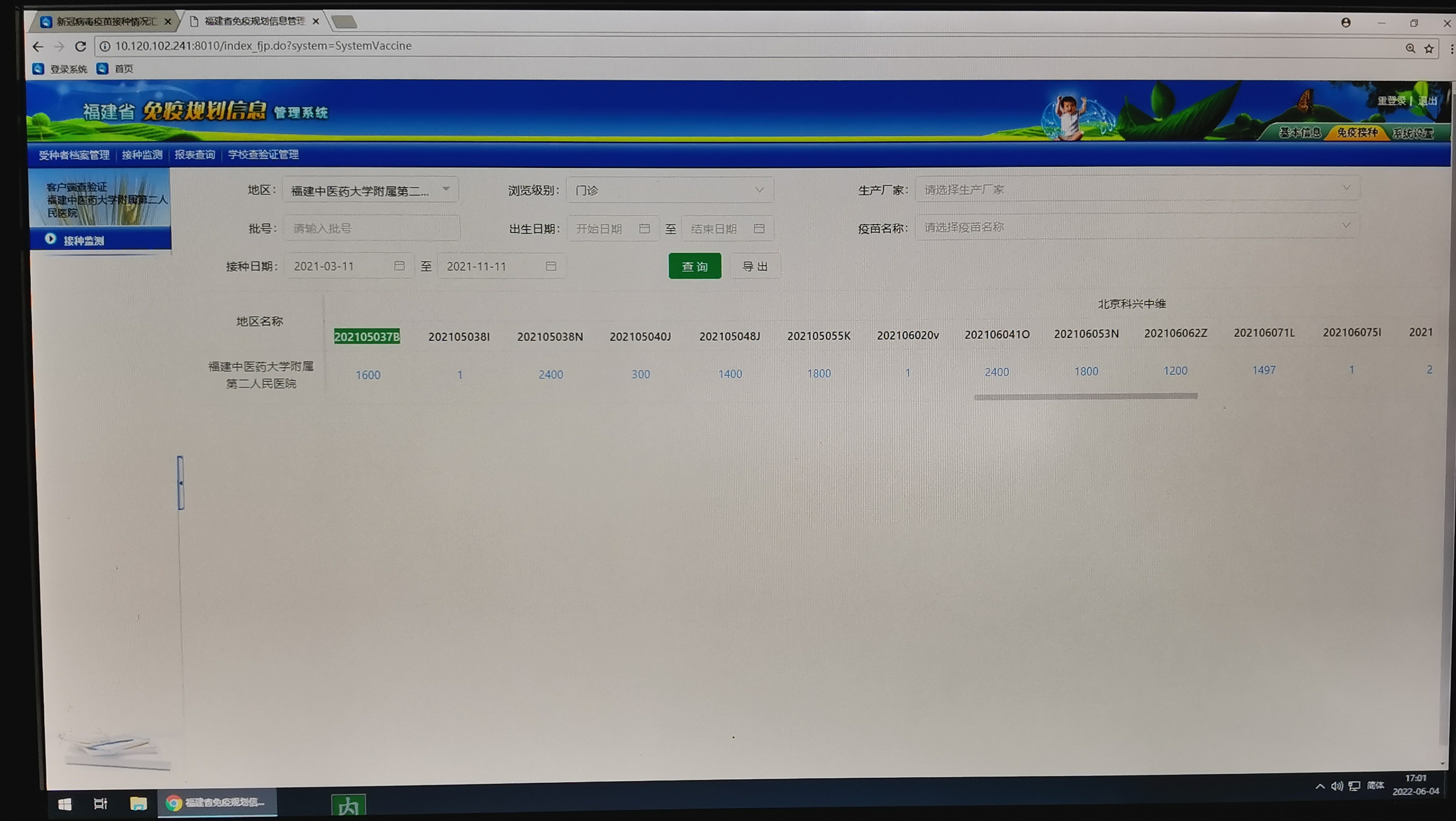

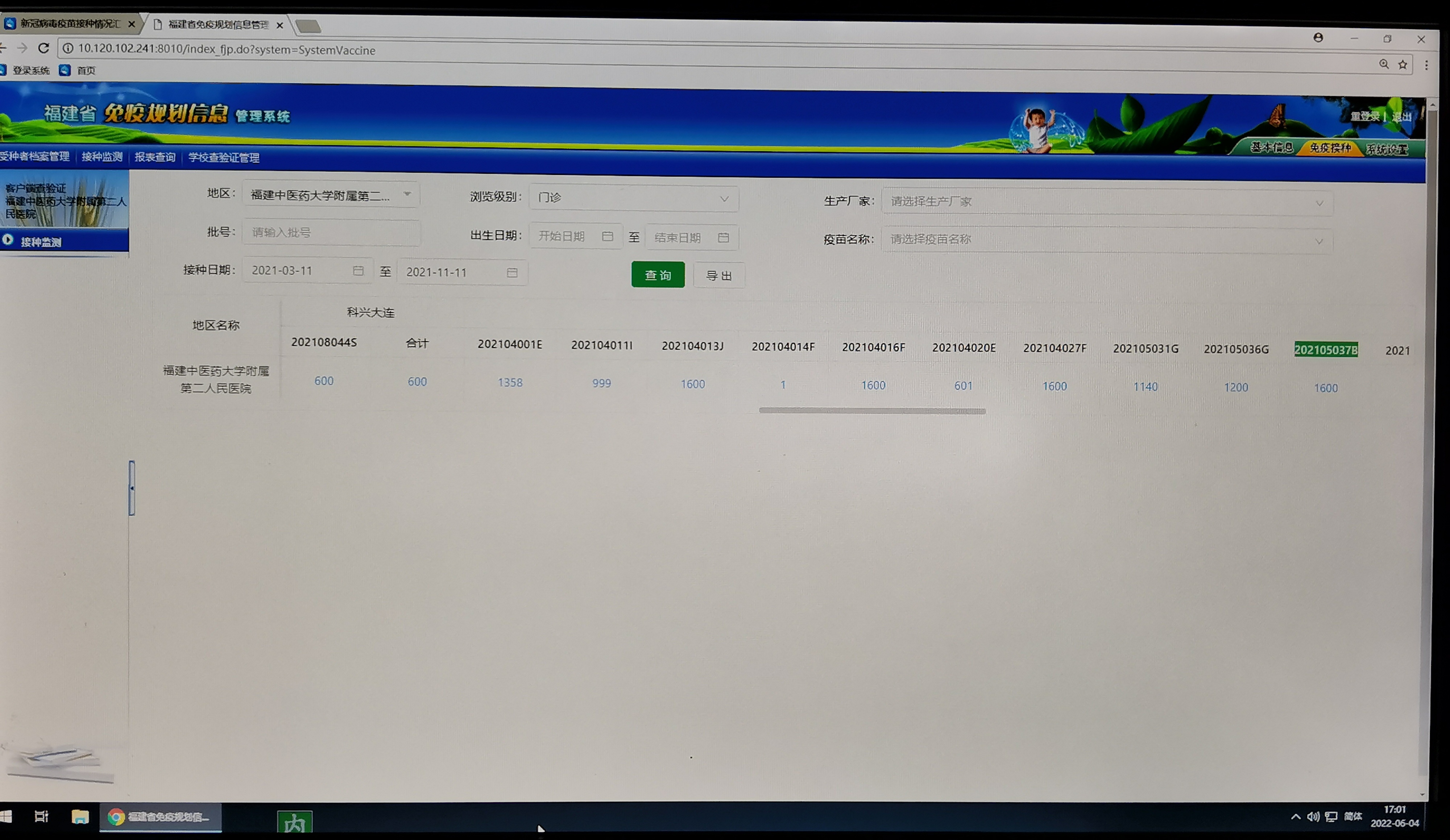

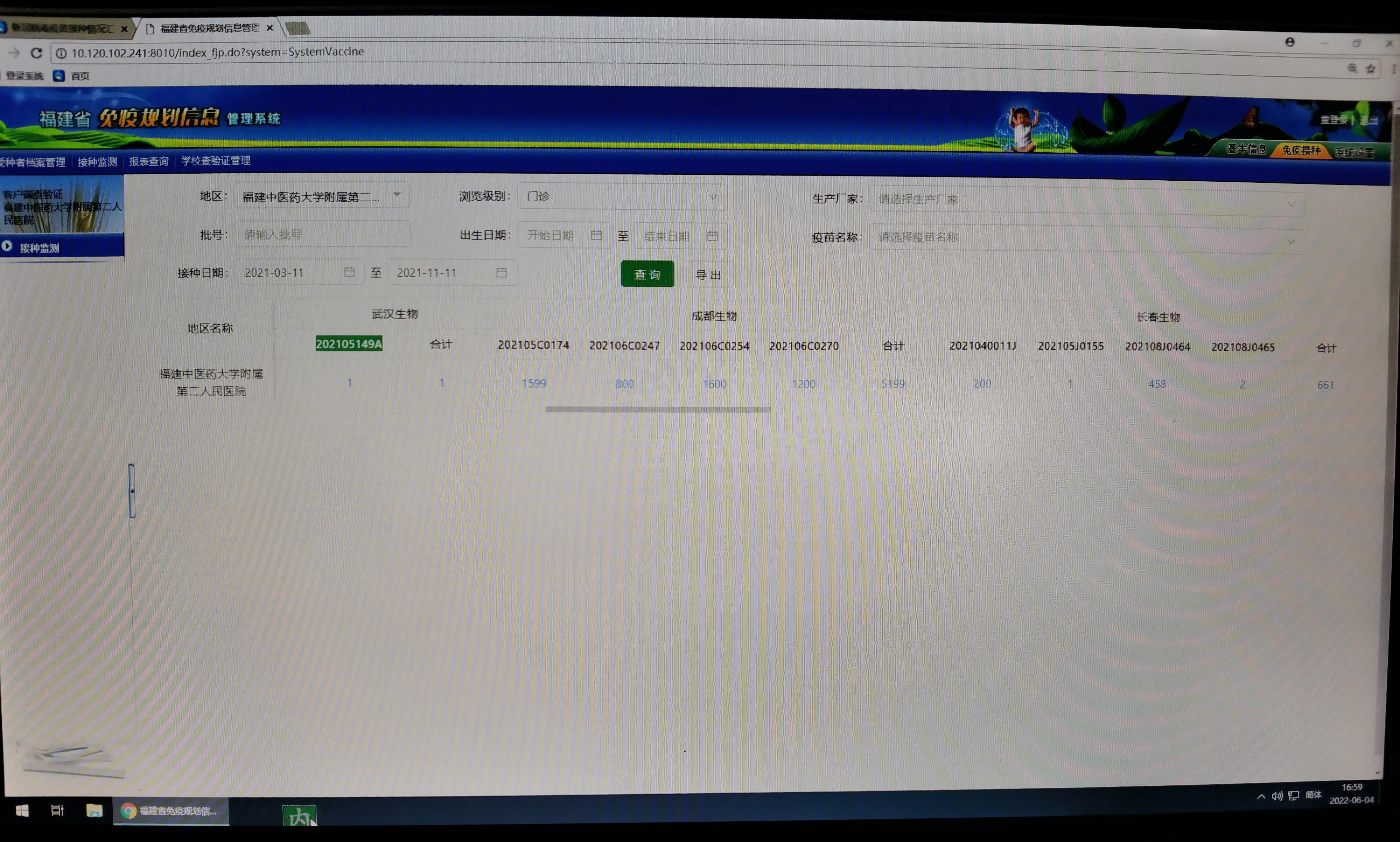

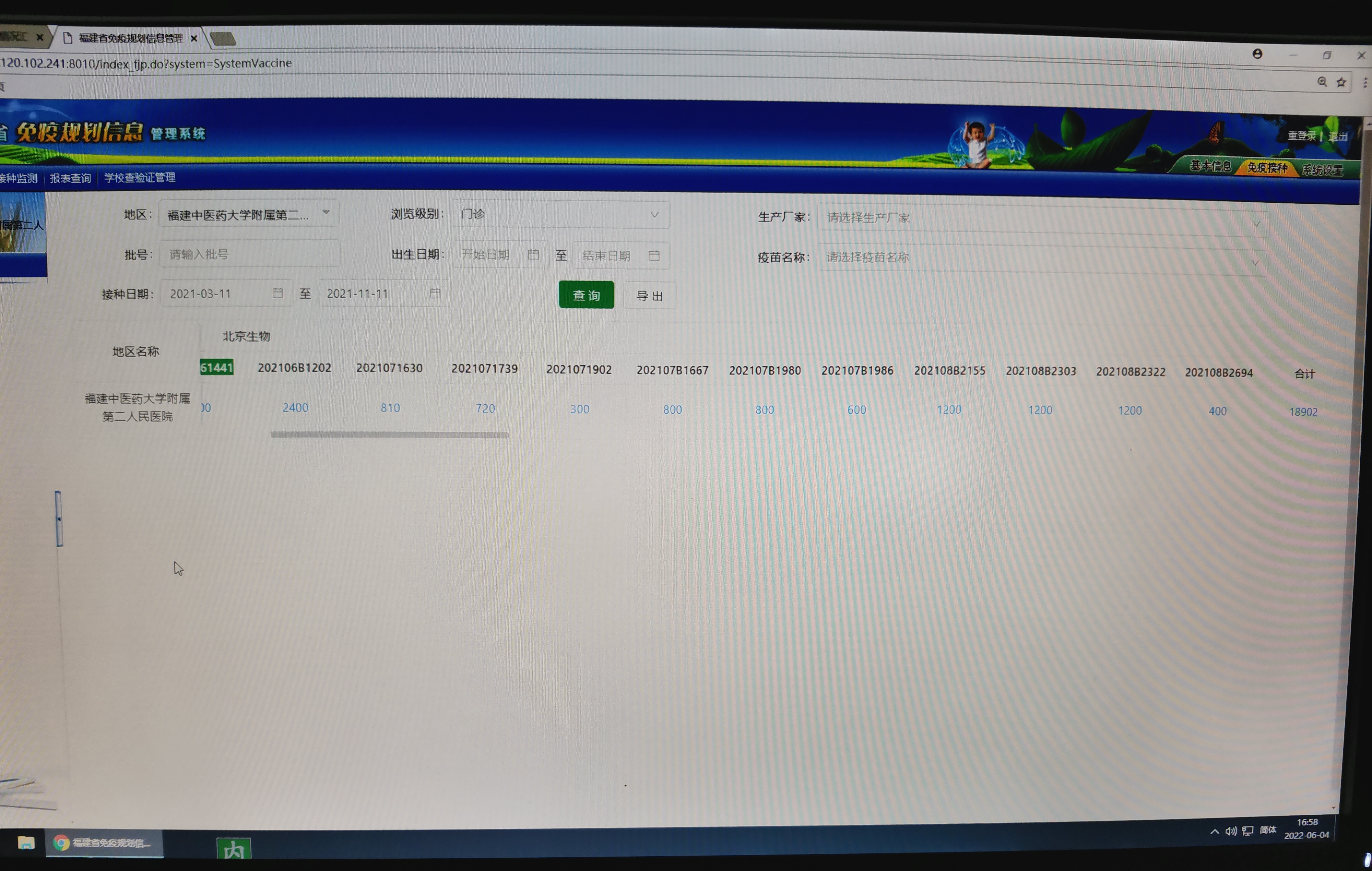


**Fig.2** Monthly number of vaccinations


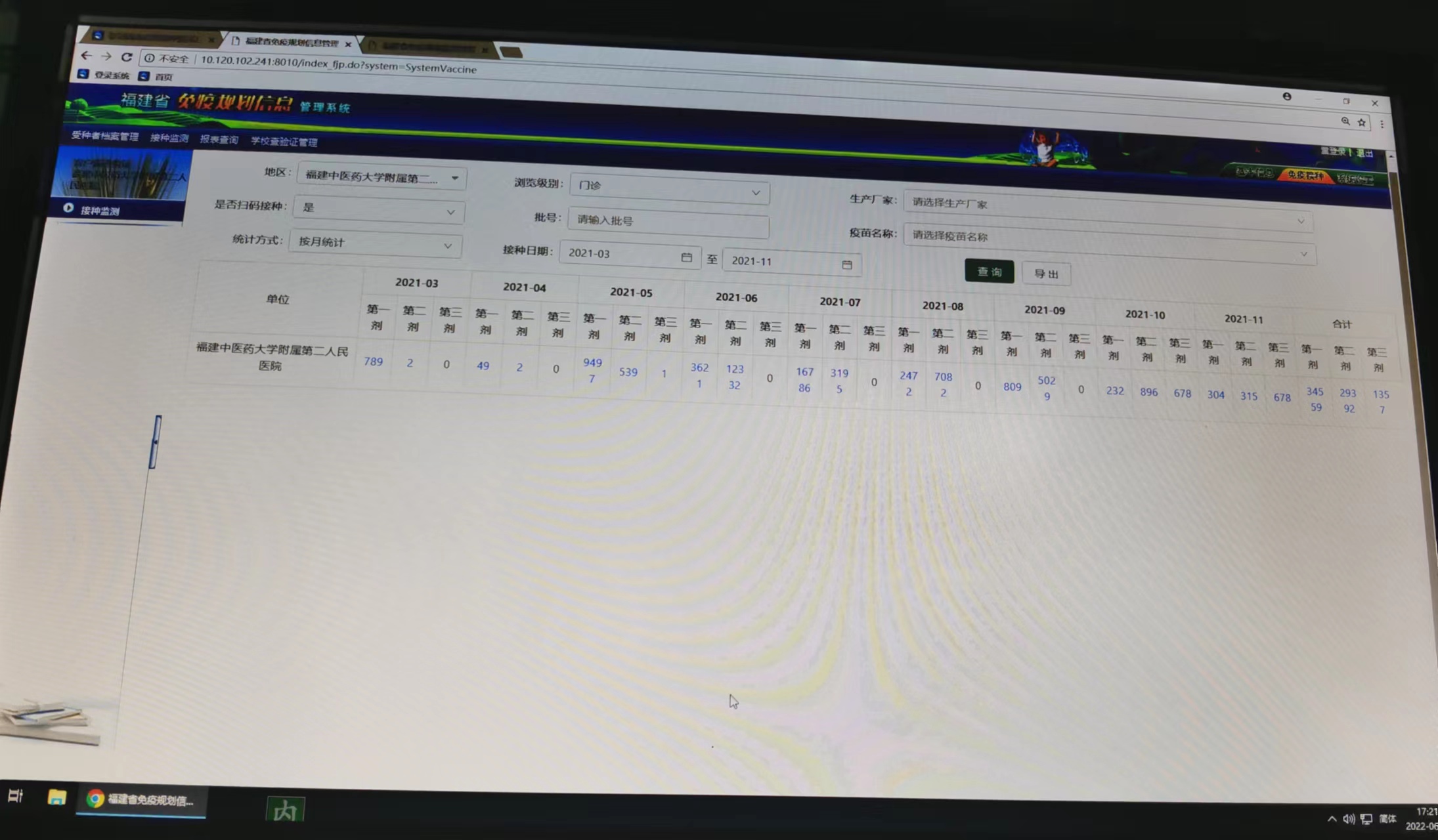


**Fig.3** Occupational classification data of COVID-19 vaccine recipients


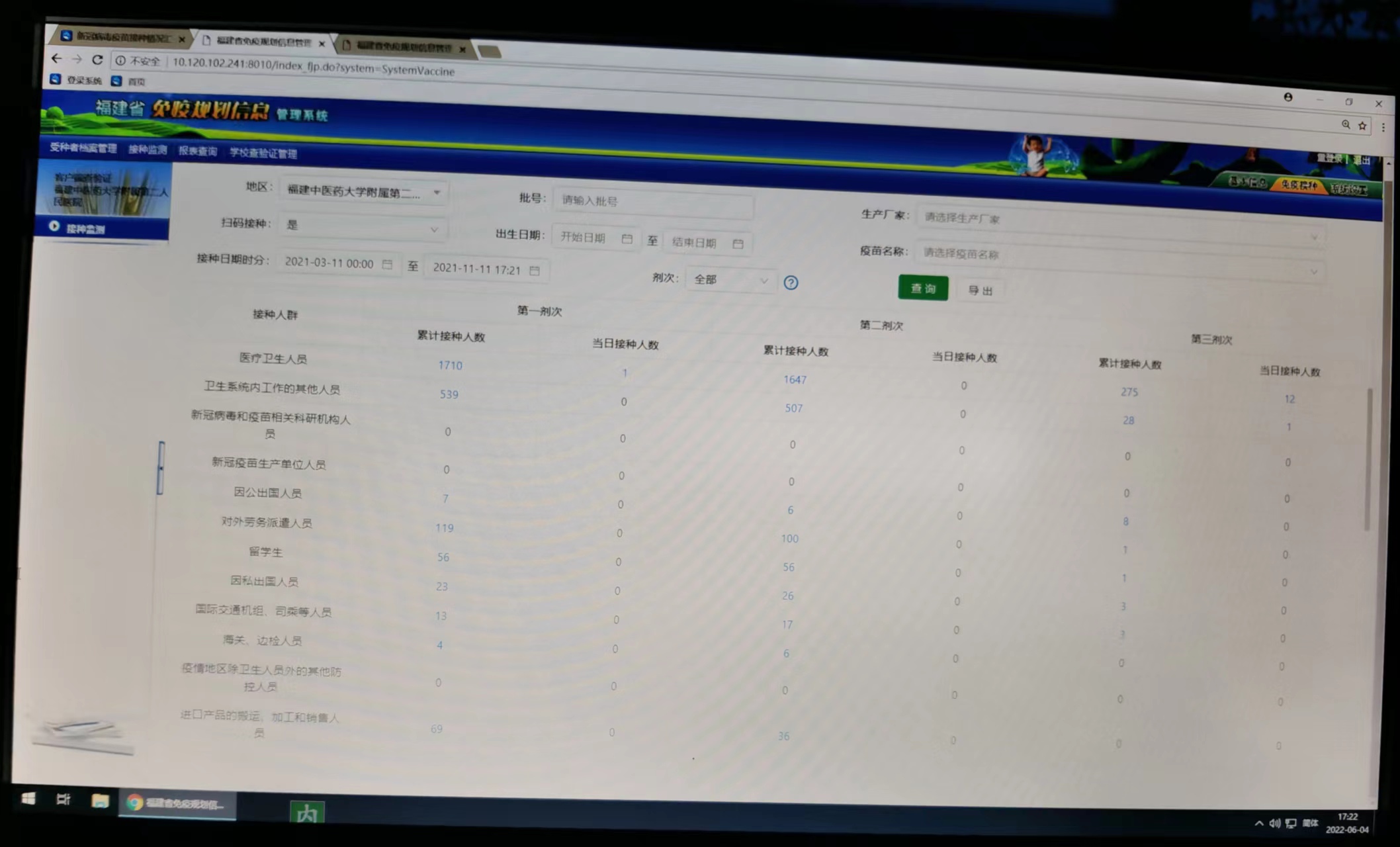

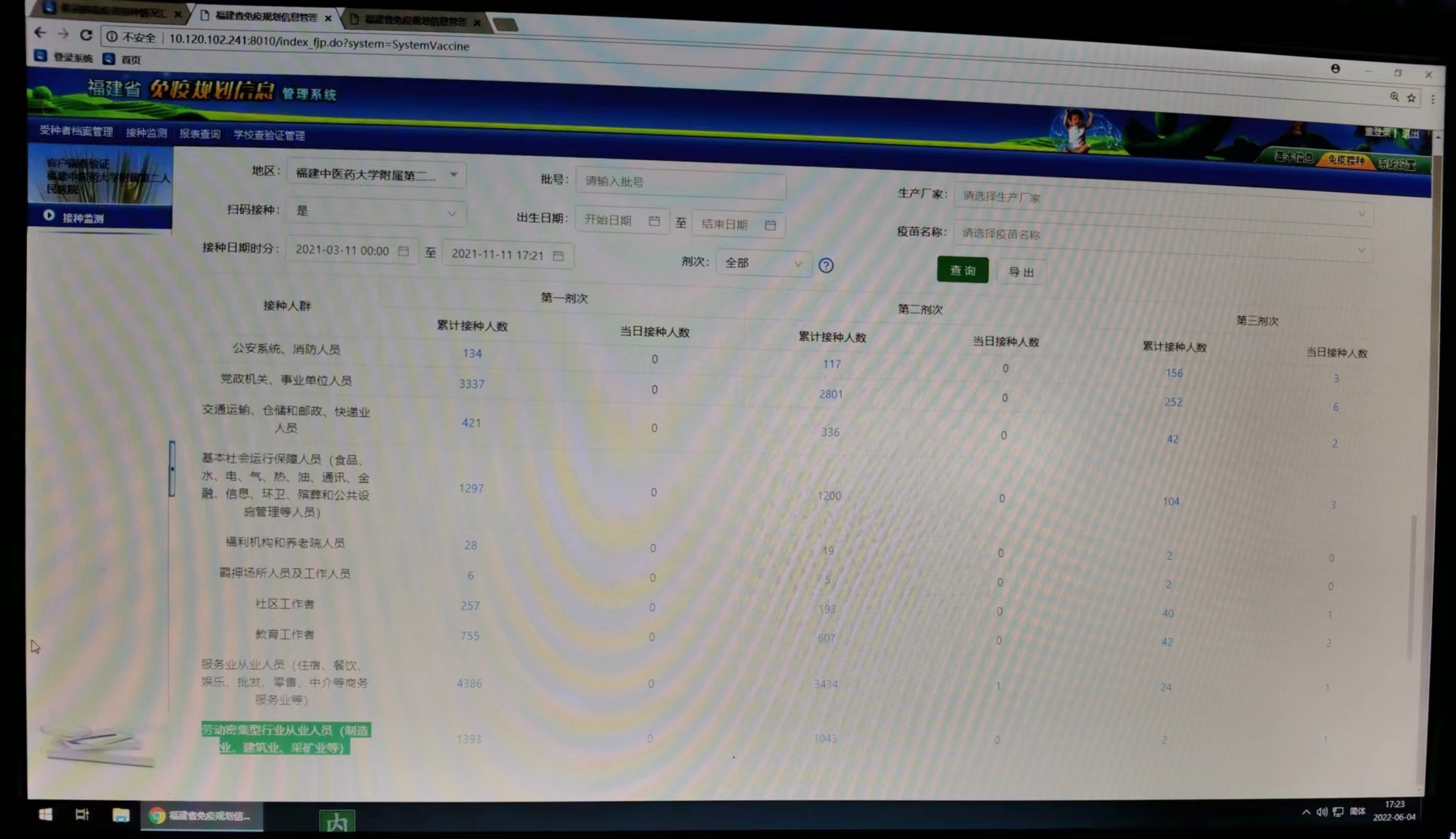

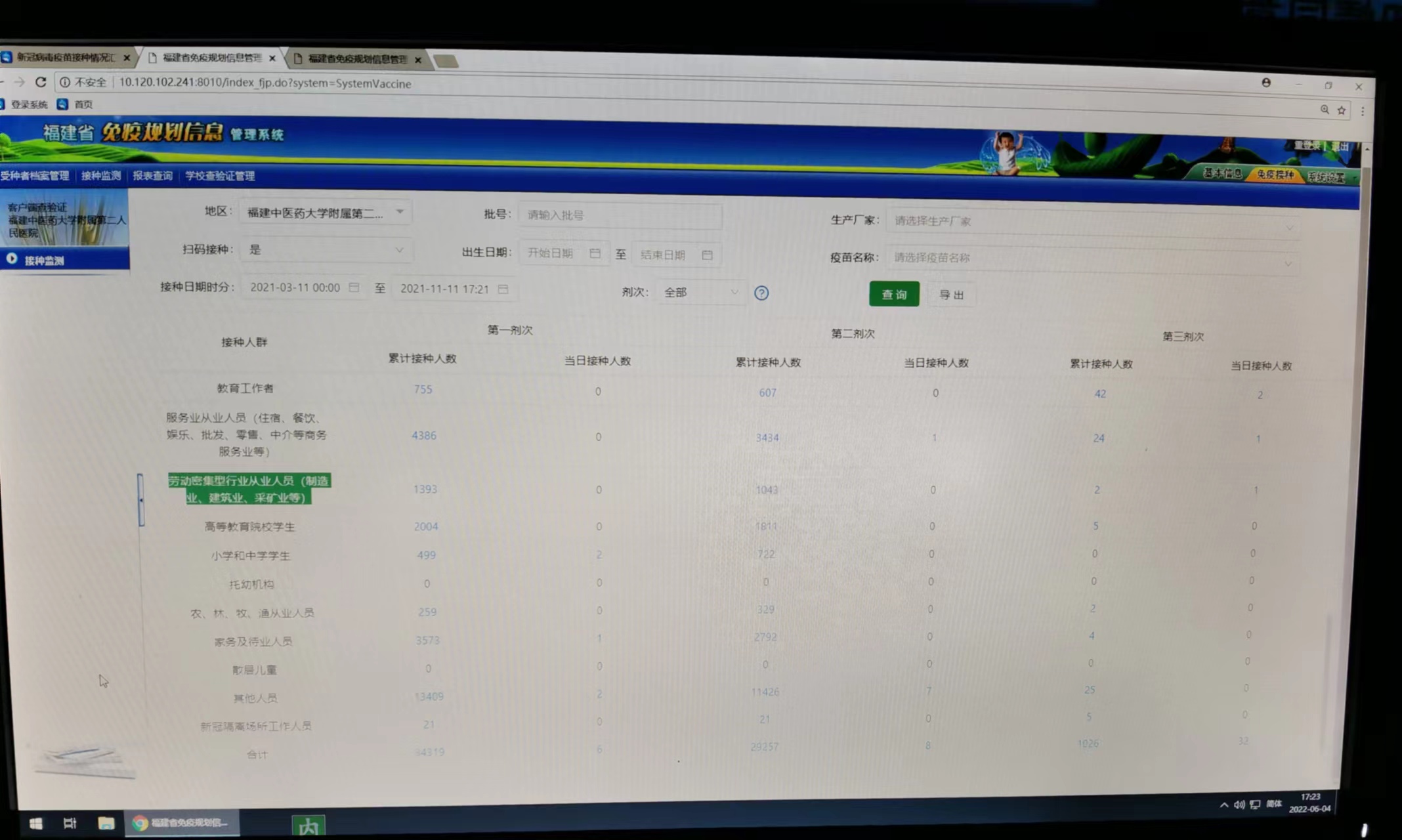


**Fig.4** Number of vaccines by age group


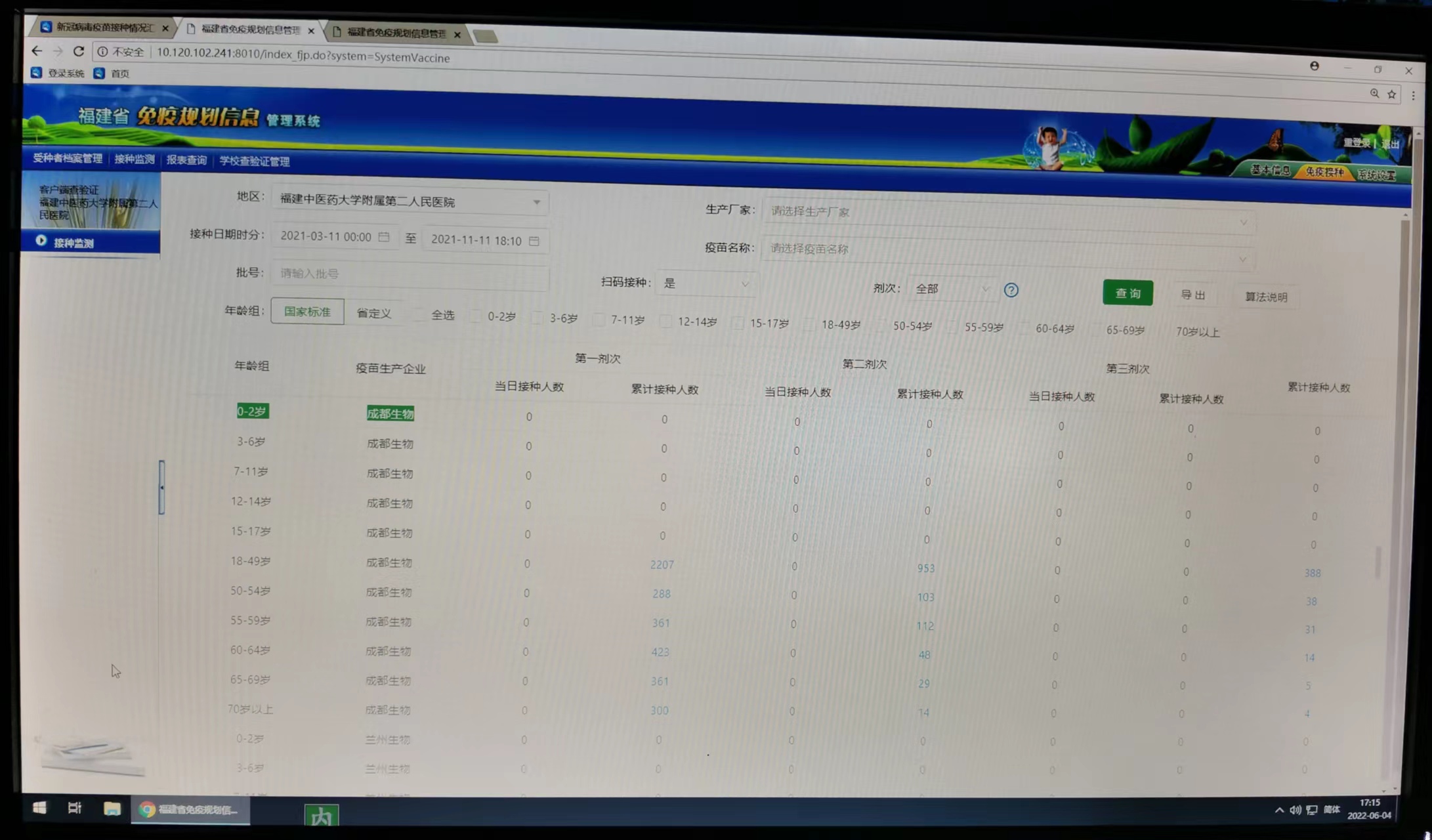

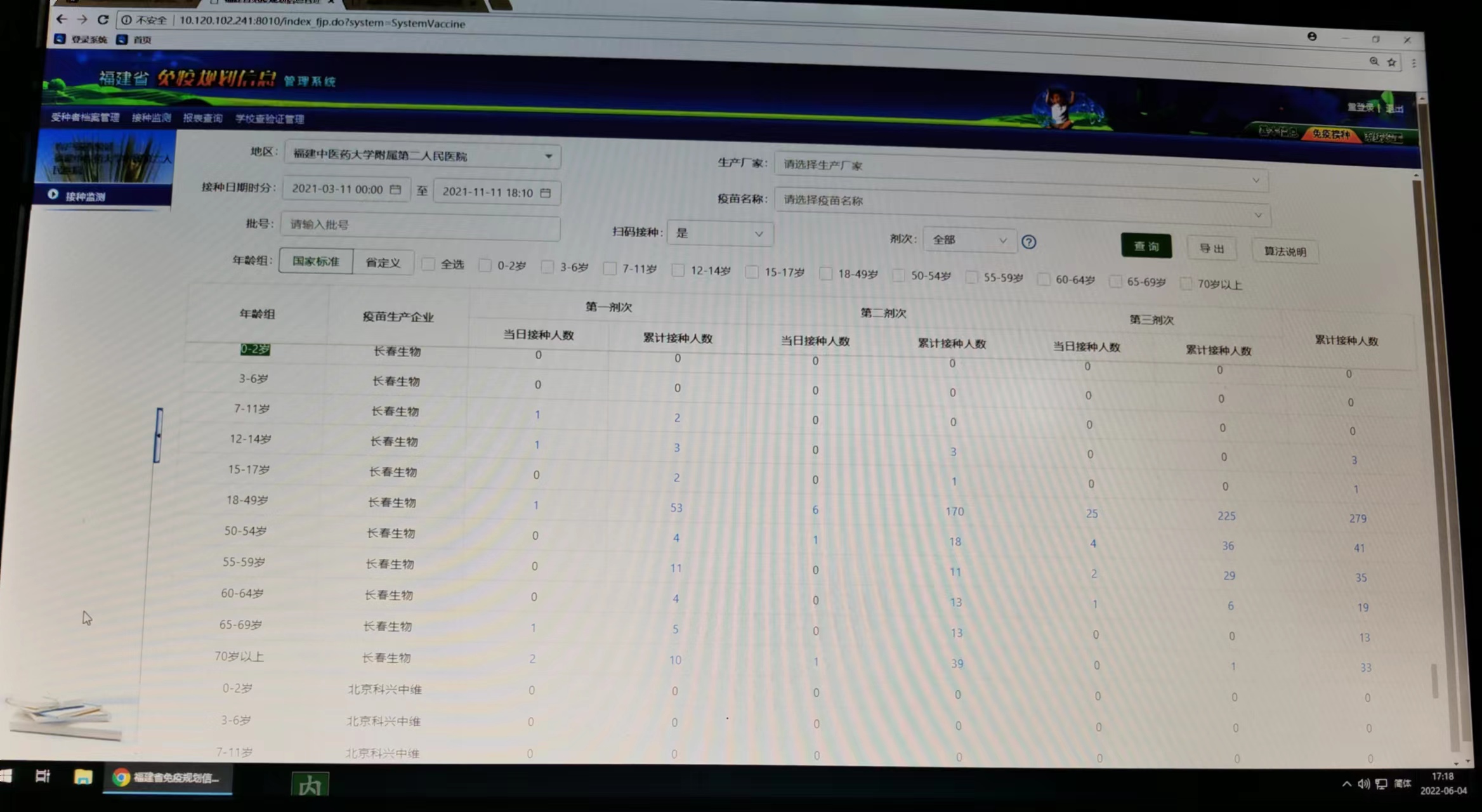

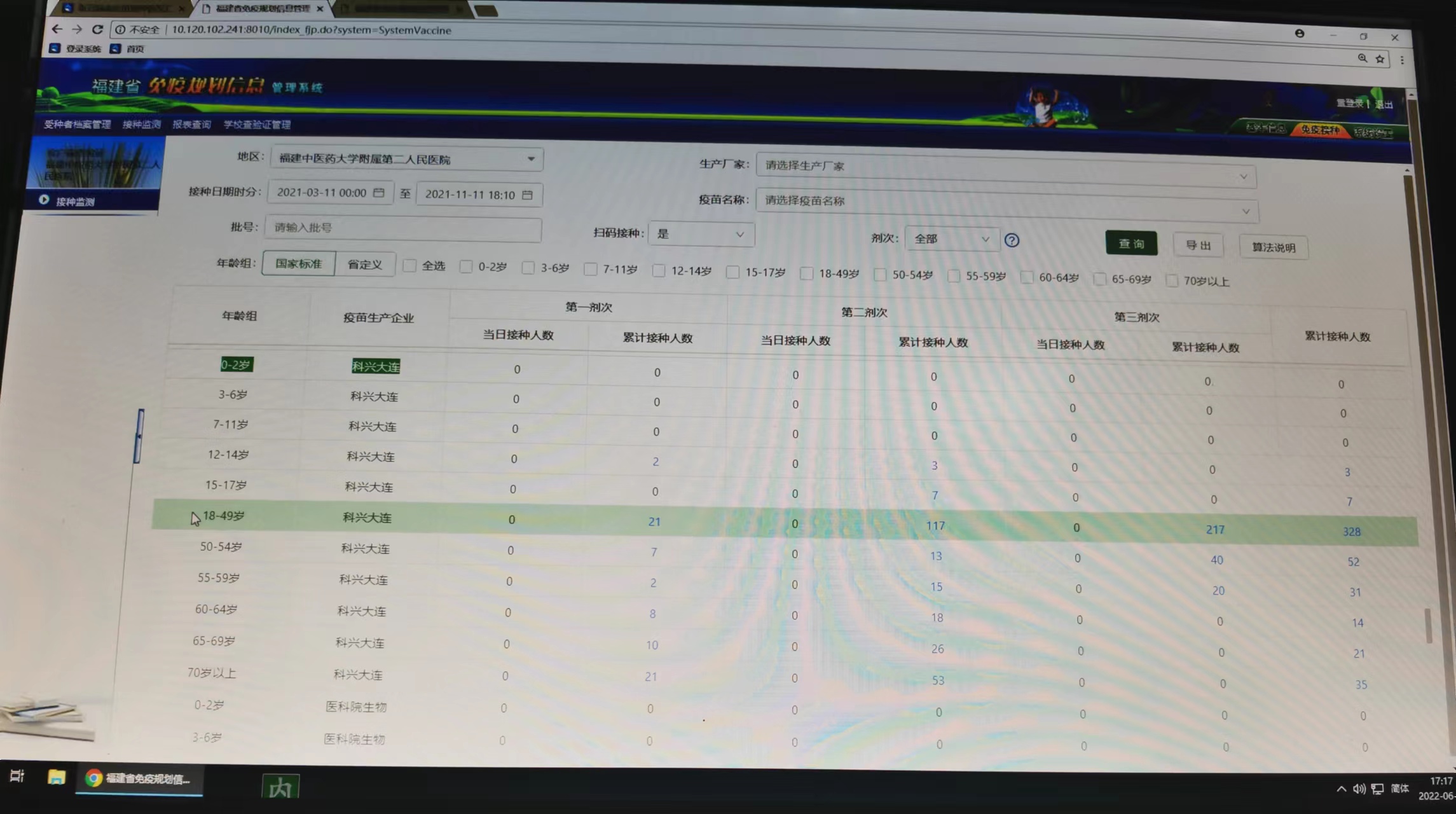

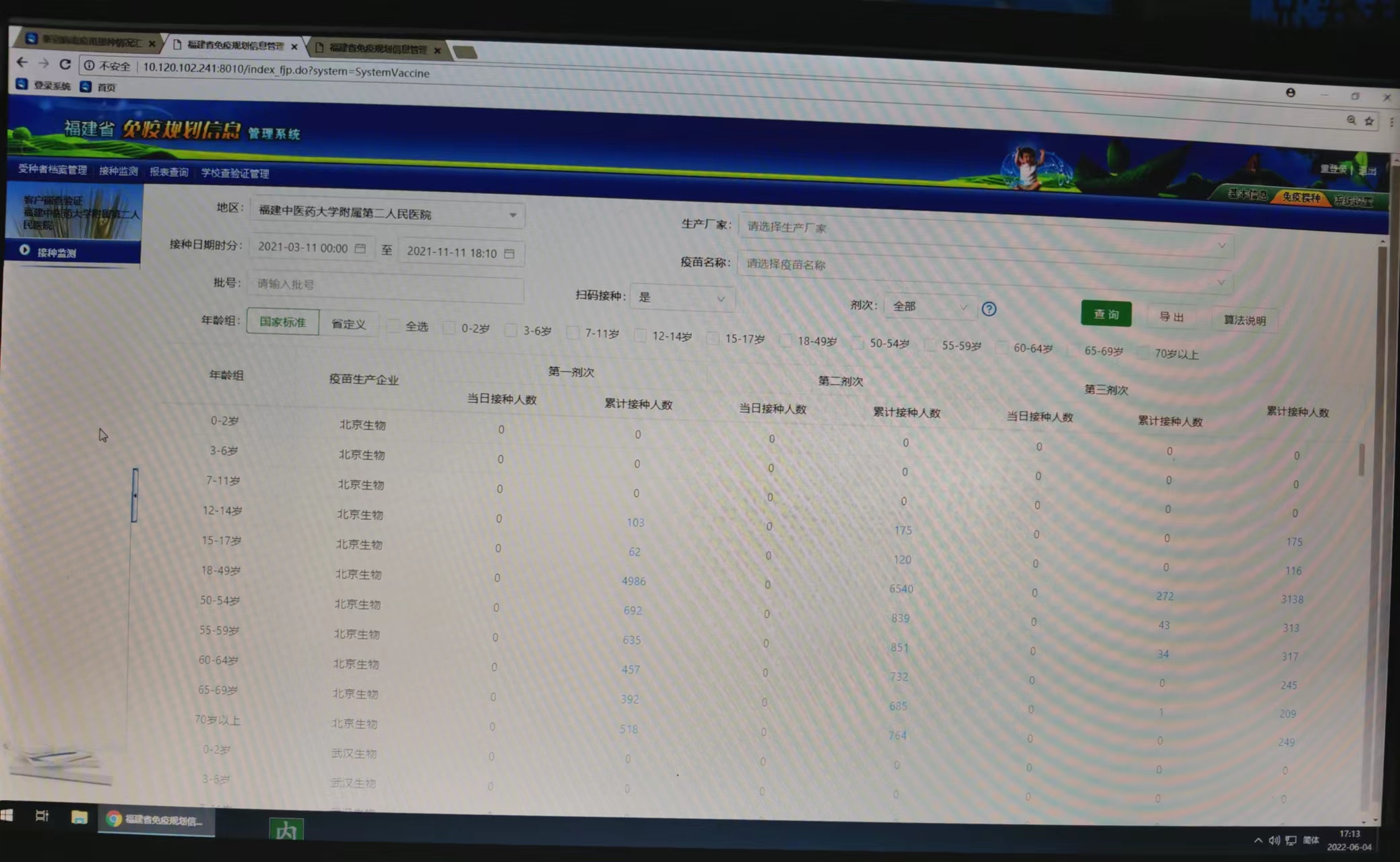

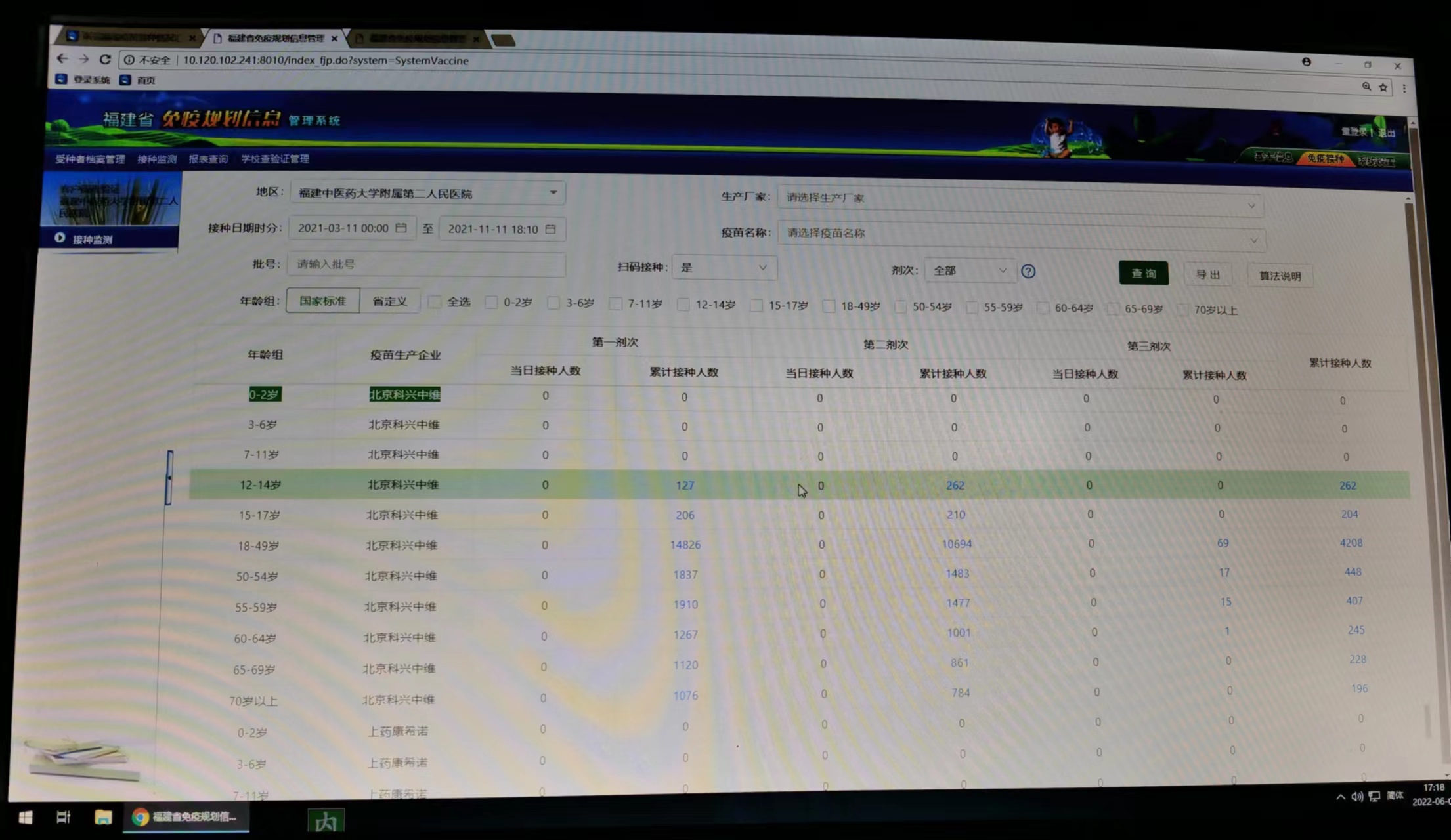

Supplement: Supplementary file 1 [file S1935789322002178sup001.docx]
